# The Topography of Poverty [Response to Letter]

**Published:** 2008-03-15

**Authors:** James B Holt

**Affiliations:** Division of Adult and Community Health, Centers for Disease Control and Prevention, Atlanta, Georgia

## In Reply:

Dr Buttery's letter (1) contains excellent observations, and he is entirely correct in his assessment of the suitability of my article (2) for presentation to policy makers. Policy makers and the lay public are not expected to have an interest in, or to comprehend, the quantitative methods behind the spatial analysis of poverty. The primary purpose of my Tools and Techniques article was to illustrate some quantitative approaches to visualization and exploratory data analysis using data available in the Community Health Status Indicators database. The article also included observations about the variable of interest: poverty. I have been surprised, but not displeased, that most feedback on this article (which I have received informally through e-mails and conversations) has focused on its substantive findings and not its methodology.

Dr Buttery's main point centers on the question of how findings of such an analysis can be effectively presented to policy makers and community members. An effective presentation should be a top priority for scientists preparing manuscripts for dissemination to nonscientific audiences such as policy makers and community members. Mapmakers have a special challenge: they are never certain who will use the finished product and for what purpose. If I were to rewrite my article for an audience of policy makers, I would reduce both the complexity and volume of quantitative jargon to an absolute minimum, while assuring the audience that the findings and conclusions are scientifically valid, backed by statistical analysis, and not merely the author's opinions based on visual interpretation of the maps alone.

The subjective nature of visual interpretation was demonstrated by the first four maps in the article (Figures 1, 2, 3, 4), which used the same data set but illustrated different spatial patterns. The spatial patterns were based on data classification methods that do not account for the spatial locations of each county's poverty rate. Spatial analytic methods are important for quantifying spatial relationships in the data set so that valid statements can be made about geographical patterns that may appear through visual interpretation of maps; the minutiae of spatial analytic methods are not so important.

For nonscientific audiences, including policy makers, the following language may help interpret Figure 5, which features spatial outliers and concentrations of high and low poverty: "Statistical techniques allow us to identify counties whose poverty rates are dissimilar to the poverty rates of their neighboring or nearby counties; these counties are referred to as spatial outliers. The same techniques allow us to identify counties whose poverty rates are similar to the poverty rates of neighboring or nearby counties; these areas represent concentrations of high poverty and low poverty. Using these techniques, we can distinguish statistically valid geographic patterns in the poverty data that may not be apparent from a visual interpretation of the mapped data alone."

A unique advantage of maps is that they simplify and illustrate patterns not discernable through data tables. Maps have visual impact; mapmakers want map readers to remember the phenomena they see in a map, and they often want to encourage appropriate action. By using spatial statistical techniques to identify geographic patterns, mapmakers use additional information in the data set — spatial information — to ensure that the resulting patterns are not subject only to the interpretations of the map reader. Mapmakers are responsible for explaining their methods but should understand that audiences vary in their need for details.

Dr Buttery's letter reinforces a key point for mapmakers and others who present data in written or graphic format: know your audience, and present and interpret your results in a way that maximizes understanding. I thank Dr Buttery for his insightful comments.
